# Occurrence of comorbid diseases in patients after COVID-19

**DOI:** 10.25122/jml-2022-0168

**Published:** 2023-03

**Authors:** Umida Kamilova, Akbal Ermekbaeva, Nuriddin Nuritdinov, Abror Khamraev, Gulnoza Zakirova

**Affiliations:** 1 Department of Therapy, Tashkent Medical Academy, Tashkent, Uzbekistan; 2 Department of Cardiology, Republican Specialized Scientific and Practical Medical Center of Therapy and Medical Rehabilitation, Tashkent, Uzbekistan

**Keywords:** COVID-19, comorbidity, risk factors, AH – Arterial hypertension, CHD – coronary heart disease, CHF – chronic heart failure, CKD – chronic kidney disease, AF – atrial fibrillation, COPD – chronic obstructive pulmonary disease, DM – diabetes mellitus

## Abstract

The COVID-19 pandemic has highlighted the potential impact of this disease on cardiovascular morbidity and mortality. Patients with established cardiovascular (CV) disease are at increased risk of severe infection and hospital-acquired adverse outcomes. This study aimed to investigate the prevalence and characteristics of comorbidities in COVID-19 patients. We analyzed data from 220 patients who previously contracted COVID-19. Statistical analysis was performed using SPSS software. The average age of the patients was 54.6 ± 11.4 years, and arterial hypertension (AH) was the most common comorbidity, affecting 55% of patients. Obesity was observed in one-third of patients, while coronary heart disease (CHD) and coronary heart failure (CHF) were reported in 17.7% and 11.8% of patients, respectively. Chronic kidney disease (CKD), atrial fibrillation (AF), and obstructive pulmonary disease (COPD) were less common. Cardiovascular diseases, particularly AH, were the most frequent comorbidities in COVID-19 patients. Understanding the prevalence and characteristics of comorbidities in COVID-19 patients is crucial for developing appropriate management strategies and improving clinical outcomes. Our findings highlight the importance of identifying and managing comorbidities in COVID-19 patients to reduce the risk of severe COVID-19 and improve clinical outcomes.

## INTRODUCTION

The COVID-19 pandemic has presented significant challenges for healthcare professionals worldwide in promptly diagnosing infections caused by the novel coronavirus, providing specialized medical care, implementing rehabilitation programs, and developing effective strategies for secondary prevention [1]. Since the emergence of COVID-19, healthcare professionals worldwide have been gathering and discussing information about its epidemiology, clinical features, and treatment in real-time [2]. As of mid-2021, official figures suggest that the number of COVID-19 cases globally has surpassed 230 million, with a likely higher number when accounting for asymptomatic cases. Understanding the long-term consequences of COVID-19 and developing effective strategies for managing patients post-COVID is of great interest, yet many challenges remain unresolved [3]. Post-COVID syndrome, characterized by signs and symptoms that persist for more than 12 weeks without any other identifiable cause, has a significant impact on patients' quality of life, emphasizing the need for optimization or the creation of new treatment algorithms and standards [4]. One of the most pressing issues of the novel COVID-19 infection pandemic is its potential impact on cardiovascular morbidity and mortality. COVID-19 may be associated with an increased incidence of acute cardiovascular (CV) events, and patients with established CV disease are at increased risk of severe infection and hospital-acquired adverse outcomes. Given the magnitude of the spread of the virus, it is equally important to understand the long-term CV consequences of COVID-19.

This study aimed to investigate the prevalence and characteristics of comorbid conditions in patients who have experienced COVID-19 and to examine the features of the post-morbid period.

## Material and Methods

We analyzed data from 220 patients who previously contracted COVID-19. Patients were included in the study 4-6 months after the onset of COVID pneumonia. The study population consisted of 107 men (48.6%) and 113 women (51.4%), with a mean age of 54.6±11.4 years. We used multi-slice computed tomography (MSCT) to assess the degree of lung damage in each patient, categorizing them into four groups: mild (54.5% of patients), moderate (32% of patients), severe (25.3% patients), and very severe (3.2% patients).

We used the Microsoft Office Excel-2020 package and SPSS software for the statistical processing of the data. Both parametric and non-parametric indicators were evaluated, and we calculated the mean value (M) and standard deviation (SD). We assessed the reliability of the data using Student's t-test and performed correlation analysis using the Pearson correlation coefficient. A significance level of P<0.05 was used to indicate statistically significant results.

## RESULTS

The data analysis revealed that arterial hypertension (AH) was the most common comorbidity among the patients, with 121 (55%) patients having this condition. One-third of the patients had obesity, while 39 (17.7%) had coronary heart disease (CHD), and 26 (11.8%) had coronary heart failure (CHF). Chronic kidney disease (CKD), atrial fibrillation (AF), and obstructive pulmonary disease (COPD) were less common comorbidities. A detailed breakdown of the comorbidities observed in the patient population is provided in Table 1.

**Table 1:** Clinical characteristics of patients observed 6 months after hospitalization.

Diagnosis of concurrent illnesses as risk factors	6 months after COVID-19 (%)
**Arterial hypertension**	55
**Obesity**	33.6
**Coronary heart disease**	17.7
**CHF**	11.8
**Atrial fibrillation**	4.1
**CKD**	6.4
**Diabetes mellitus**	14.1
**COPD**	4.5

Many patients with COVID-19 continued to experience a range of health problems after being discharged from the hospital. A significant proportion of patients, 36.6% and 25.7%, respectively, reported at least one symptom after three and six months. Weakness was the most prevalent symptom, reported by 31.8% and 24.1% of patients at three and six months, respectively. Shortness of breath was also a common complaint, reported by 28.6% and 17.9% of patients at three and six months, respectively (Table 2). Notably, 18.1% of patients experienced an increase in blood pressure within the first three months, despite receiving previously effective antihypertensive therapy, and 11.6% reported palpitations. Other less common symptoms included guarded chest pains and loss of taste and smell.

**Table 2:** Persistent symptoms in the post-hospital period.

Symptoms	After 3 months (n=220)	After 6 months (n=212)
**Weakness**	70 (31.8%)	51 (24.1%)
**Dyspnea**	63 (28.6%)	38 (17.9%)
**High blood pressure**	40 (18.1%)	39 (18.3%)
**Heartbeat**	26 (11.6%)	11 (5.2%)
**Cough**	18 (8.2%)	8 (3.7%)
**Chest pain**	9 (4.1%)	7 (2.8%)
**Loss of sense of smell (anosmia) or taste (ageusia)**	5 (2.3%)	1 (0.47%)

14. 5% of patients experienced shortness of breath with vigorous exercise, 8.2% with moderate exercise, 5% with light exercise, and 1.4% at rest after 3 months of rehabilitation. Patients with cardiovascular pathology were most frequently affected by dyspnea six months later. After 6 months, 13 patients (6.1%) continued to experience shortness of breath with severe physical activity, and 3.8%, 2.3%, and 0.5% reported dyspnea with normal physical exercise, slight physical exertion and at rest, respectively.

Additionally, among patients with newly emerging diseases after three and four to six months, hypertension was the most common, accounting for 2.3% and 2.8% of new diseases, respectively (Table 3).

**Table 3:** Newly diagnosed diseases in the post-COVID period.

	After 3 months (n=220)	After 6 months (n=212)
**Arterial hypertension**	5 (2.3%)	6 (2.8%)
**Ischemic heart disease**	1 (0.45%)	3 (1.4%)
**Myocardial infarction**	1 (0.45%)	2 (0.9%)
**CHF**	2 (0.9%)	3 (1.4%)
**Atrial fibrillation**	1 (0.45%)	1 (0.45%)
**CKD**	1 (0.45%)	0
**Diabetes mellitus**	3 (1.4%)	1 (0.45%)
**Stroke**	1 (0.45%)	0

Furthermore, this study revealed an increase in the proportion of patients with "new" coronary artery disease four to six months after the initial COVID-19 diagnosis, with a prevalence of 1.4%, compared to 0.45% at three months. The incidence of new cases of myocardial infarction (MI) was also higher in the four to six-month period compared to the first three months. A similar trend was observed for new cases of coronary heart failure (CHF), with a prevalence of 0.9% in the first three months and 1.4% in the four to six-month period.

## DISCUSSION

The incidence of comorbid conditions in post-COVID-19 patients observed in our study was generally consistent with the occurrence of diseases in the patient population of the same age [5,6]. This finding is in agreement with data from other observational studies of patients in the post-hospital period, such as the study by Günster et al. [7], which identified arterial hypertension (AH), diabetes mellitus (DM), cardiac arrhythmias, chronic kidney disease (CKD), and coronary heart failure (CHF) as the most common comorbidities in patients discharged from the hospital. In line with these results, the AKTIV registry reported long-term persistence of symptoms in 38.2% of patients who underwent COVID-19. Similar to our study, Huang et al. [8] found that fatigue or muscle weakness (63%), sleep problems (26%), and the presence of anxiety and/or depression (23%) were the most common persistent symptoms in 1733 patients observed for six months after hospital discharge. According to the National Institute for Health and Care Excellence (NICE) guidelines for post-COVID syndrome [9], about one in five people who tested positive for COVID-19 had symptoms that lasted 5 weeks or longer, and one in ten people had symptoms that lasted 12 weeks or longer. Most often, patients complained of chronic cough, shortness of breath, chest tightness, cognitive dysfunction, and extreme fatigue [10,11]. Recent studies have extensively investigated tachycardia in patients after COVID-19 [12]. Ståhlberg et al. introduced the term "post-COVID-19 tachycardia syndrome" to describe the presence of tachycardia in post-COVID syndrome, a specific phenotype of "post-acute COVID-19 syndrome", which is defined as symptoms persisting for 4-12 weeks or longer than 12 weeks after COVID-19 infection [13]. Thus, in patients included in the ACTIV registry, comorbid diseases comparable to those cited above were identified, leading to increased readmissions and mortality in the posthospital period [14,15]. Apparently, the dominant nature of cardiovascular diseases in patients in the post-hospital period of COVID-19 is universal in all regions of the world and raises questions about the possible affinity of the virus to certain tissues and organs.

## CONCLUSION

Our study found that cardiovascular diseases were the most common comorbidities among patients who underwent COVID-19. This is consistent with previous studies and highlights the importance of monitoring and managing these patients to address potential long-term health consequences. The emergence of the post-COVID syndrome and the persistence of symptoms in a significant proportion of patients further emphasize the need for continued research and development of effective strategies for prevention and treatment. Overall, the COVID-19 pandemic has highlighted the significant impact of infectious diseases on global health, underscoring the importance of ongoing efforts to improve public health infrastructure, preparedness, and response.

## Personal thanks

The authors would like to thank the patients who aided in conducting the present study.
